# CANDLE/Supervisor: a workflow framework for machine learning applied to cancer research

**DOI:** 10.1186/s12859-018-2508-4

**Published:** 2018-12-21

**Authors:** Justin M. Wozniak, Rajeev Jain, Prasanna Balaprakash, Jonathan Ozik, Nicholson T. Collier, John Bauer, Fangfang Xia, Thomas Brettin, Rick Stevens, Jamaludin Mohd-Yusof, Cristina Garcia Cardona, Brian Van Essen, Matthew Baughman

**Affiliations:** 1 Argonne National Laboratory, Argonne, IL USA; 2 Los Alamos National Laboratory, Los Alamos, NM USA; 3 Lawrence Livermore National Laboratory, Livermore, CA USA; 4 Minerva, San Francisco, CA USA

**Keywords:** Sample, Article, Author

## Abstract

**Background:**

Current multi-petaflop supercomputers are powerful systems, but present challenges when faced with problems requiring large machine learning workflows. Complex algorithms running at system scale, often with different patterns that require disparate software packages and complex data flows cause difficulties in assembling and managing large experiments on these machines.

**Results:**

This paper presents a workflow system that makes progress on scaling machine learning ensembles, specifically in this first release, ensembles of deep neural networks that address problems in cancer research across the atomistic, molecular and population scales. The initial release of the application framework that we call CANDLE/Supervisor addresses the problem of hyper-parameter exploration of deep neural networks.

**Conclusions:**

Initial results demonstrating CANDLE on DOE systems at ORNL, ANL and NERSC (Titan, Theta and Cori, respectively) demonstrate both scaling and multi-platform execution.

## Background

Cancer is an extremely complex disease, which disrupts basic biological processes at a fundamental level, leading to renegade cells threatening the health of the individual. Fortunately, with major technological advances in molecular sequencing, molecular and cellular imaging, and high-throughput screening techniques, it is now possible to probe the complexity of the disease at an unparalleled level, which provides a window into the behavior of the disease at unprecedented time and spatial scales. The application of these technologies has produced massive datasets that can be analyzed with automated machine learning (ML) techniques.

Simultaneously, the development of post-petascale and near-exascale computers is ongoing. Top tier computers in the U.S. include ALCF Theta, OLCF Titan, and NERSC Cori. These systems feature extremely large node counts (thousands to tens of thousands), and are equipped with nodes of many integrated cores or accelerator technologies, such as GPUs. These systems also have large hierarchical memory and I/O resources. Thus, they are capable of performing machine learning workloads that would be extremely time-consuming to run elsewhere (on open science infrastructure).

This work offers an early attempt to apply these top-tier systems to three problems in cancer research. We focus here on the problem of hyperparameter optimization, which tries to find high performing configurations for neural networks. The design parameters broadly include the number of layers, neurons per layer, activation function, and so on. The quality of the network is essentially its accuracy; a loss function *F* is determined such that its value is a measure of the error in the trained network behavior when applied to a validation set. The hyperparameter optimization problem is to minimze *F*(*p*), for all parameter sets *p* in the valid parameter space *P*, however, *P* is large and *F* is expensive. *P* is the cross product of all valid network settings, some of which may be categorical, some integer, some continuous. Evaluating *F* involves training the network on a training data set and applying it to the validation set.

This problem is an excellent but challenging candidate for workflow technologies, because it involves running a large number of independent tasks, each of which communicates only with the optimization algorithm. Each task is capable of utilizing all the compute resources on the node, as the available 3rd-party ML implementations are multi-threaded or deployable on a GPU. The tasks run for minutes, hours, or longer. Workflow systems would be challenged, however, by the scale and complexity of the large-scale resources that we desire to apply to the problem. Also, we desire to apply complex 3rd-party algorithms written in Python or R to control this workflow by driving an optimization loop. Similarly, because the ML algorithms are written in C/C++ with complex Python-based interfaces, there is a software interfacing challenge. Additionally, we must collect data on *F* during the run, as well as various other data for profiling or validation.

Success in the application of ML to cancer research will enable and greatly accelerate the capabilities needed to realize the promise envisioned for the ‘Cancer Moonshot’ [1] and establish a new paradigm for cancer research for years to come by making effective use of the ever-growing volumes and diversity of cancer related data to build predictive models, provide better understanding of the disease and, ultimately, provide guidance and support decisions on anticipated effective treatments for individual patients.

**Contributions** This paper offers the following: 1) A description of several machine learning-based workflows relevant to cancer. 2) An architecture for coordinating and storing workflow processes and data products. 3) Performance results from running these workflows on large-scale systems.

The remainder of this paper is organized as follows. In the remainder of this section, we describe the aspects of machine learning relevant to this work. In “Methods” section, we describe the architecture of the CANDLE/Supervisor software system, and the three workflows currently supported by CANDLE/Supervisor, and the practicalities and portability issues. In “Results” section, we describe performance results from these systems. In “Discussion” section, we describe future work, and we conclude in “Conclusions” section.

### The CANDLE project

Machine learning (ML) has the capability to transform many scientific problems. In response to the growing power of ML techniques and the increasing available computing power at large scale computing facilities, the U.S. Department of Energy Exascale Computing Project (ECP) launched the **Cancer Distributed Learning Environment (CANDLE)**. CANDLE is developing a suite of software to support scalable deep learning on DOE supercomputing resources. While the CANDLE project is explicitly aimed at supporting deep learning in the three cancer pilot projects in the near-term, its longer-term goal is to support a wide variety of deep learning applications across DOE science domains.

### Frameworks for machine learning

Deep learning frameworks are under active development by diverse research communities in both industry (Google, Facebook, Microsoft, etc.) and academia (Berkeley, Oxford, Toronto, etc.). These include Caffe [2], Keras [3], Theano [4], Torch [5], Poseidon [6], Neon [7], TensorFlow [8], CNTK [9], and the Livermore Big Artificial Neural Net (LBANN) [10]. Each of these frameworks differ with respect to the machine learning tasks they target, their ease of use, data pre-processing, and target problems. Most frameworks were architected for a single node implementation and a few distributed memory multi-node implementations have recently emerged; but these implementations are primarily targeted at smaller core counts and for commodity cluster environments. Moreover, these implementations rely on avoiding communication by storing data on local disks. Implementations targeting high-performance computing systems will need novel techniques to fully exploit the system interconnect bandwidth and topologies, as well as the deep memory hierarchies.

### Hyperparameter search

The simplest, though most costly, methods for hyperparameter optimization include exhaustive space search (the brute force method), simple gradient descent, multiple gradient descent, and random search. Though these search algorithms can be tuned to execute quickly (random search) or to find the optimal solution (exhaustive search), the marginal optimization with respect to utilized resources is not efficient for problems with *O*(10^9^) or greater reasonable discrete parameter combinations. There are two primary drawbacks to utilizing an *a priori* user-specified set of discrete hyperparameters for reducing loss: 1) it requires the user to make assumptions concerning topological efficiencies and efficacies and 2) it is limited to a small, finite set of models (i.e., it is forcing a complex algorithm into constrained bounds). By including effective reductions possible using gradient descent, we may gain one or two orders of magnitude of search space, however, this is still well below the *O*(10^21^) complexity that is possible in the current CANDLE workflows.

Currently, several frameworks and libraries exist to accelerate model exploration. As described in “Methods” section, we use the EMEWS [11] framework to directly incorporate parameter exploration methods for efficient exploration of order >10^9^ spaces. This framework uses the Argonne-developed Swift/T [12, 13] language to distribute the model exploration workload efficiently across a multi-node system.

HyperTune [14] uses Bayesian optimization to refine given network hyperparameters. This implementation of Bayesian optimization excels as it does not require calculation of many multidimensional derivatives. The algorithm can be thought of as finding direction from a random sample – a set of hyperparameters is chosen, then another, and if the second is a better set than the first, the algorithms aims in that direction. This method excels as it is extensible and can find reasonable solutions with much less compute time than evolutionary algorithms but it can also “bounce around,” not settling on a given set of values or displacing itself past a promising minima.

Another alternative is the popular Python library, SciKit-Learn [15]. This is a multipurpose machine learning library for Python (easily integrated with Keras) and can be used for hyperparameter search. HyperOpt [16] is a hyperparameter search framework that is designed to perform searches using distributed hardware. HyperOpt has a SciKit-Learn variant [17].

Another approach is evolutionary algorithms. One of the most prominent and robust implementation of genetic algorithms for hyperparameter search is the NeuroEvolution of Augmenting Topologies (NEAT) algorithm [18]. The NEAT method begins by spawning a genome and then producing a population based on that genome. Using a selection function, the algorithm then usually removes the least fit (those with the highest error rate or loss) members of the population, then uses crossover between members of the remaining subpopulation to produce the next generation. It does this on two levels, both within each node (neuron) and with the topology of the network. Using this genetic-style algorithm, one is often able to find a robustly effective solution. There are, however, some drawbacks of the NEAT algorithm (or, at least, its specific “NEAT-python” [19] implementation). The primary factor that would most limit us in our application is NEAT‘s alteration of intra-node weights and parameters. While this can definitely prove beneficial by reducing loss at the “starting point” of training, it also serves as a topology specific feature that somewhat precludes comparison of pure topological strengths and weaknesses. The other limiting factor is the overhead required to generate the network from scratch.

Another system for evolutionary algorithms for hyperparameter tuning is Optunity [20], a DEAP-dependent [21] hyperparameter tuning engine. DEAP (Distributed Evolutionary Algorithms in Python) is a Python framework that implements different, generalized evolutionary algorithms. Optunity acts as an interface between DEAP and the network to be optimized, allowing for easy deployment of these various algorithms for the purpose of hyperparameter optimization. Optunity is an excellent implementation of evolutionary algorithms for the purpose of hyperparameter tuning, however, it was last updated nearly one year ago (2016).

mlrMBO [22] is a R package for model-based approaches developed for tackling expensive black-box optimization by approximating the given objective function through a surrogate regression model. It is designed for optimization problems with mixed continuous, categorical and conditional parameters. mlrMBO follows Bayesian optimization [23] approach which proceeds as follows. In the initialization phase, *n*_*s*_ configurations are sampled at random, evaluated, and a surrogate model *M* is fitted with the input-output pairs. In the iterative phase, at each iteration, *n*_*b*_ promising input configurations are sampled using the model *M*. These configurations are obtained using infill criterion that guides the optimization and tries to trade-off exploitation and exploration. The infill criterion selects configurations that either have a good expected objective value (exploitation) or high potential to improve the quality of the model *M* (exploration). The algorithm terminates when user-defined maximum number of evaluations and/or wall-clock time is exhausted.

In this work, we focused on mlrMBO as it was shown to obtain state-of-the-art performance on a wide range of test problems, where it was benchmarked against other approaches such as DiceOptim, rBayesianOptimization, SPOT, SMAC, Spearmint, and Hyperopt. Crucial to the effectiveness of mlrMBO is the choice of the algorithm used to fit *M* and the infill criterion. Given the mixed integer parameters in the hyperparameter search, we used random forest [24] because it can handle such parameters directly, without the need to encode the categorical parameters as numeric. For the infill criterion, we used the qLCB [25], which proposes multiple points with varying degrees of exploration and exploitation.

## Methods

Emerging multi-petaflop supercomputers are powerful platforms for ensembles of neural networks that can address many problems in cancer research, but it is difficult to assemble and manage large studies on these machine, which have tens of thousands of compute nodes. Typical workflow approaches would face challenges due to system scale, system complexity, management of complex workflow patterns, integration with disparate software packages, and data acquisition. CANDLE/Supervisor addresses the problem of hyperparameter optimization for cancer-based problems, and solves the common workflow challenges outlined above.

**Fig. 1:**
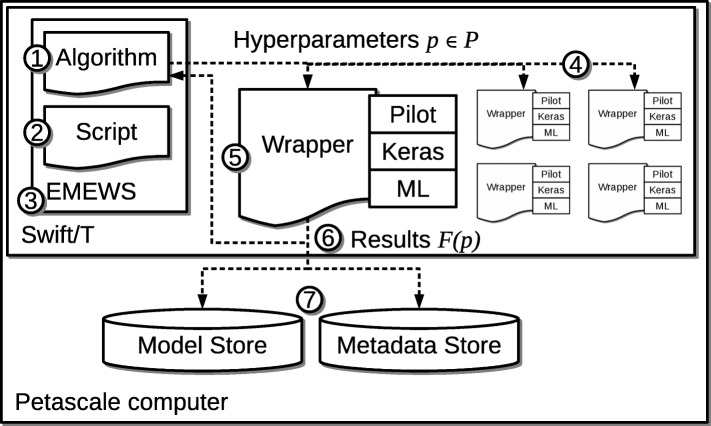
CANDLE/Supervisor overall architecture

To support the search patterns described in “Background” section, we developed the CANDLE/Supervisor architecture diagrammed in Fig. 1. The overall goal is to solve the hyperparameter optimization problem to minimize *F*(*p*), where *F* is the performance of the neural network parameterized by *p*∈*P*, where *P* is the space of valid parameters.

The optimization is controlled by an **Algorithm** 1 O selected by the user. The Algorithm can be selected from those previously integrated into CANDLE, or new ones can be added. These can be nearly any conceivable model exploration (ME) algorithm that can be integrated with the **EMEWS** 3 O software framework. EMEWS [11] enables the user to plug in ME algorithms into a workflow for arbitrary model exploration; optimization is a key use case. The ME algorithm can be expressed in Python or R. This is implemented in a reusable way by connecting the parameter generating ME algorithm and output registration methods to interprocess communication mechanisms that allow these values to be exchanged with Swift/T. EMEWS currently provides this high-level queue-like interface in two implementations: EQ/Py and EQ/R (EMEWS Queues for Python and R). The Algorithm is run on a thread on one of the processors in the system. It is controlled by a Swift/T script 2 O provided by EMEWS, that obtains parameter tuples to sample and distributes them for evaluation.

The Swift/T [12, 13] workflow system is used to manage the overall workflow. It integrates with the various HPC schedulers “Computing systems” section to bring up an allocation. A Swift/T run deploys one or more load balancers and many worker processes distributed across compute nodes in a configurable manner. Normally, Swift/T evaluates a workflow script and distributes the resulting work units for execution across the nodes of a computer system over MPI. Swift/T can launch jobs in a variety of ways, including in-memory Python functions in a bundled Python interpreter, shell commands, or even MPI-based parallel tasks. However, in this use case, workflow control is delegated to the Algorithm via the EMEWS framework, which provides the Swift/T script.

During an optimization iteration, the Algorithm produces a list of parameter tuples 4 O that are encoded as arguments to a Python-based **Wrapper** script 5 O. These wrapper scripts are the interfaces to the various CANDLE Pilot applications. The parameters are encoded in JavaScript Object Notation (JSON) format which can be easily converted by the Python **Wrapper** script into a Python dictionary, from which a CANDLE Pilot application can retrieve the parameter values. These scripts are run concurrently across the available nodes of the Swift/T allocation, typically one per node. Thus, the **ML** has access to all the resources on the node. The ML is the underlying learning engine; we have tested with Theano and TensorFlow. The **Pilots** are Python programs that implement the application-level logic of the cancer problem in question. They use the **Keras** interface to interact with the ML and are coded to enable the hyperparameters to be inferred from a suitable default model file, or to be overwritten from the command line. It is this construction that allows the parameter tuples to be easily ingested by the respective Pilots, and use a standardized interface developed as part of the project.

The result of a Wrapper execution is a performance measure on the parameter tuple *p*, typically the validation loss. Other metrics could be used, including training time or some combination thereof. These are fed back to the Algorithm by EMEWS to produce additional parameters to sample. The results are also written to a Solr-based **Metadata Store** 7 O, which contains information about the Wrapper execution. The Metadata Store accesses are triggered by Keras callback functions, which allow Wrapper code to be invoked by Keras at regular intervals. Thus, a progress history is available for each learning trial run, as well as for the overall optimization workflow. Good models can also be selected and written to a **Model Store**.

### Workflows

In this section, we describe how the framework described in “Methods” section is applied to the three pilot cancer problems. CANDLE is investigating three promising pilot applications of ML technology to cancer research:

**P:RAS – The RAS pathway problem.** The RAS/RAF pathway is a series of chemical events that is implicated in 30% of cancers. The goal of this pilot is to understand the molecular basis of key protein interactions in this pathway.

**P:DRUG – The drug response problem.** The goal of this pilot is to develop predictive models for drug response that can be used to optimize pre-clinical drug screening and drive precision medicine based treatments for cancer patients.

**P:TREAT – The treatment strategy problem.** The goal of this pilot is to automate the analysis and extraction of information from millions of cancer patient records to determine optimal cancer treatment strategies across a range of patient lifestyles, environmental exposures, cancer types and healthcare systems.

While each of these three challenges are at different scales (i.e., molecular, cellular and population) and have specific scientific teams collaborating on the data acquisition, data analysis, model formulation, and scientific runs of simulations, they also share several common threads. They are all linked by common sets of cancer types that will appear at all three scales, all have to address significant data management and data analysis problems, and all need to integrate simulation, data analysis and machine learning to make progress. We have focused on the machine learning aspect of the three problems and, in particular, we are focused on building a single, scalable deep neural network computing environment to support them.

### P:RAS – The RAS pathway problem

For this Pilot the goal is to develop a predictive capability for modeling the behavior of proteins on membranes and to apply that capability to RAS and effector proteins along the primary RAS signaling pathways. We expect that as a result of this capability we will accelerate the identification and development of effective therapeutics targeting cancers driven by RAS mutations, including the three deadliest cancers occurring today: pancreatic, lung and colon. By exploiting a mixture of atomistic and coarse-grained resolutions we anticipate modeling for the first time a relevant size (*O*(10^10^) atoms) and time-scale (*O*(10^9^) timesteps) to allow investigation of targetable binding sites along the RAS signaling cascade. Unfortunately, the combinatorial number of possible binding interactions along the cascade renders a human-guided exploration of the state-space unlikely to uncover a site suitable for therapeutic intervention. What is required is a formalism for defining and following a path of simulations that will lead us to a targetable site.

The starting point for our deep learning is the output of these extremely large-scale molecular dynamics calculations. We aim to use unsupervised learning to uncover features from these simulations that can be used to describe the state-space of protein movement and binding in a higher level model. These higher level models can then be used to explore (far more efficiently) the possible dynamics of RAS interactions, delivering many millions of hypothetical trajectories which can be scored according to likelihood. By investigating (through direct numerical simulation) the most likely of these trajectories, we “close the loop” — essentially testing our hypothesis and then learning from the results. Any new information is used to refine the definitions of likelihood and affect future hypothesis. This combination of machine learning and molecular dynamics to develop and test hypotheses of protein binding will dramatically enhance our understanding of RAS signaling pathways (potentially leading to a cure) and demonstrates a new and powerful way to use high performance computing as tool for scientific discovery.

**Pilot application.** The P:RAS pilot is a two-stage set of stacked autoencoders that learn both molecular- and frame- level features for the coarse-grained molecular dynamics simulation of a lipid membrane. The first part of the neural network is a multi-stage stacked, convolutional autoencoder with a local receptive field sized to observe individual molecules, which produces molecular-level features. The second part of the neural network is a multi-stage, stacked fully connected autoencoder that processes the output of the molecular autoencoder to create a compressed representation of the entire simulation frame. For preliminary network optimizations, we have explored the following hyperparameters: 1) number of convolutional layers and features in the molecular autoencoder, 2) number of fully-connected layers and size of each layer, and 3) size of stochastic gradient descent mini-batch.

### P:DRUG – The drug response problem

Our ultimate goal is to fully exploit exascale computing to develop the predictive models necessary to guide the choice of drug treatment for a tumor based on that patient’s molecular biomarkers and knowledge of drug responses in other cases. The development of CANDLE will bring deep learning to bear on this problem at an unprecedented scale, and we believe will produce models uniquely capable of making precision medicine a reality. Deep learning has the potential to generate models that take into account a vastly increased diversity of input features than other types of machine learning [26]. Today machine learning is typically used to estimate drug response and patient outcome from one type of molecular data such as gene expression; however it has been demonstrated that models that incorporate more than one type of molecular information can be more accurate [27]. Our goal in this problem is to develop a scalable deep learning framework that will support the utilization of many types of information as input to models. Ideally, we will integrate into our models information about drug molecular structures, drug interactions, drug combinations and drug molecular targets with information about the patient’s genetics, including their baseline genotype as well as the specific genetics and other molecular and cellular properties of their tumor, including gene mutations, gene expression patterns, proteome, transcriptome including small and non-coding RNAs, metabolomics, prior treatments, co-morbidities and environmental exposure.

Our current working data contains drug and drug-like molecular screening data from over 300,000 compounds that have been tested on at least 60 cell lines giving us *O*(10^7^) training cases. For each tumor derived cell line, we have molecular characterization data that includes many types of microarrays each with ∼10^5^ data points; we have genetic variation data for these sample that consist of 10^7^ single nucleotide polymorphisms (SNPs); variety of proteomics, metabolomics, and transcription datasets including over 50,000 types of small and non-coding RNAs. For the compounds involved in screening, we can compute molecular characterization data (e.g., drug descriptors and molecular fingerprints) that when taken together are *O*(10^6^) features per molecule. Thus, our initial deep learning formulation of the drug response problem has an input data volume of between 10^14^−10^15^ measurements or approximately 1PB. The ten-year problem target is at least an order of magnitude larger than this. To our knowledge, this would be one of the largest deep learning problems in biomedical science. One of the largest training sets in the DNN community is a 15TB image recognition dataset [28]. Our ten-year goal is to expand this capability by at least an order of magnitude (10PB input data), requiring between 100TB and 1PB of high-speed memory for a single network instantiation and with a target training epoch runtime of hours.

**Pilot application.** The P:DRUG is a binary classification task on 1400 RNA-seq based gene expression profiles from the NCI Genomic Data Commons (GDC). 700 of these samples are from tumor tissues and the other 700 are their matched normals. There are 60,483 features for each sample that are fed into a neural network with a default configuration of two dense layers on top of two convolution layers. The following hyperparameters are explored to optimize our network architecture: 1) learning rate, 2) batch size, 3) number of epochs, 4) dropout, 5) activation function, 6) loss measure, 7) optimizer, 8) the number of convolution layers and the number of neurons in each convolution layer, and 9) the number of dense layers and the number of neurons in each dense layer.

### P:TREAT – The treatment strategy problem

Our goal is to exploit exascale computing to develop the predictive models necessary for population-wide cancer surveillance that extends beyond the clinical trial setting. The treatment strategy problem tackles the critical issue of clinical translation to determine to what extent scientific advances, such as those made within the RAS pathway and drug response problems, translate successfully in the real world. The treatment strategy problem requires integration of heterogeneous datasets as well as deep analytic techniques to understand the interrelationships among genetic, lifestyle and environmental factors in patient-specific cancer etiology and cancer outcomes.

To achieve our overarching goal, we will first leverage the CANDLE environment to deploy deep learning for automated extraction of clinical variables about patient-level cancer management trapped in unstructured text data from daily clinical practice. These variables capture important information about the patient’s cancer staging, administered therapies, disease progression (i.e., recurrence, metastasis), and outcome. Such information is critical to understand the impact of cancer treatment strategies and policies in the broad population as part of the national cancer surveillance program. Current practice relies on manual information extraction, an approach that is neither scalable nor comprehensive for a variety of reasons; the number of people living with cancer increases (roughly 15,000,000 people live with cancer in the US [29], new diagnostic and therapeutic biomarkers are continuously introduced, and new therapeutic options enter the clinical arena. Traditional natural language processing (NLP) algorithms have been developed to automate this process. The NLP algorithms rely on carefully crafted keyword-based rules for information extraction. With the well-known variation in clinical expression and the size of the controlled medical vocabularies containing more than 100,000 medical terms and expressions (describing diseases, conditions, symptoms, and medical semantics that are typically present in unstructured clinical text), hand-engineered rule extraction is neither scalable nor effective for large-scale clinical deployment. Deep learning has the potential to address these challenges and capture both semantic and syntactic information in clinical text without having explicit knowledge of the clinical language. However, the deep learning tools that can handle the specific requirements of this third challenge (input space (*O*(10^6^) patients) × feature space (*O*(10^5^) medical terms and expressions) × output space (*O*(10^5^) medical biomarkers and clinical endpoints throughout a cancer patient’s medical care trajectory) do not currently exist. We will develop those tools, focusing specifically on semi-supervised learning since it is impractical to collect millions of expert-annotated clinical reports. A semi-supervised algorithmic framework is best suited to this challenge, balancing carefully the number of labeled data (>10,000 clinical reports) and unlabeled data (>2,000,000 clinical reports) to be made available to us by NCI. We will explore convolutional, deep-belief, and deep-stacking networks. In addition, we will implement a multi-task deep learning framework that can be used for joint classification/information extraction tasks.

**Pilot application.** For the P:TREAT Pilot, which involves training a multi-task deep neural network (MT-DNN), we used the following hyperparameters to optimize our network architecture: 1) learning rate, 2) batch size, 3) number of epochs, 4) dropout, 5) activation function, 6) loss measure, 7) optimizer, 8) number of folds, 9) the number of neurons in the shared layer, and 10) the number of neurons in the task-specific layer. For the MT-DNN, we chose three classification tasks, namely i) primary site, ii) tumor laterality, and iii) histological grade. For each of the parameters outlined above, we run a parameter sweep on our MT-DNN to iteratively optimize the average accuracy per training task. The end of the hyperparameter sweep results in a MT-DNN that is optimally performant on the three classification tasks.

### Computing systems

The three workflows described previously “Workflows” section were run on ALCF Theta, OLCF Titan, and NERSC Cori. These systems vary greatly in their hardware and software systems. The following is an overview of their system parameters:

- **ALCF** ***Theta*** **at Argonne National Laboratory**
  - 3624 nodes with item 64-core Intel Xeon Phi item 16 GB MCDRAM, 192 GB of DDR4 RAM
  - Python 2.7.13, Keras 2.0.2, TensorFlow 1.2.0
  - Scheduler: Cobalt
- **OLCF** ***Titan*** **at Oak Ridge National Laboratory**
  - 18,688 nodes with item 16-core AMD CPU item NVIDIA Kepler K20X GPUs item 32 GB RAM
  - Python 3.6, Keras 2.0.3, TensorFlow 1.0.1
  - Scheduler: PBS
- **NERSC** ***Cori*** **at Lawrence Berkeley National Laboratory**
  - 2388 nodes with item Intel Xeon Haswell CPUs item 128 GB RAM
  - 9688 nodes with item Intel Xeon Phi item 16 GB MCDRAM, 96 GB DDR
  - Python 2.7.12, Keras 2.0.0, TensorFlow 1.2.0
  - Scheduler: SLURM

As tabulated above, it is clear that these systems vary significantly in their hardware capabilities and installed software systems. This does not include differences in compiler versions, software module management, and storage system policies or capabilities.

We use Swift/T to abstract the scheduler and compute layout settings. The launch parameters for Swift/T allow the user to specify the scheduler type, processor count, workers per node, and other common settings in a uniform way across systems.

We use our Wrapper script abstraction “Methods” section to abstract the Python configuration and ML library settings. The wrapper script is invoked in one of two ways, either by a short piece of Python code, the text of which is embedded in the Swift/T script and executed directly by the Swift/T runtime embedded Python interpreter, or by a bash script that is executed via a Swift/T app function [12]. App functions are Swift/T language functions that are implemented as command-line programs, in this case a shell script that calls the Python interpreter passing it the wrapper script as an argument. In both cases, the Swift/T script receives the hyperparameters from the model exploration algorithm and passes them to the wrapper script either via a string template in the embedded Python code or as a command line argument to the bash script.

The workflows were run on Cori using embedded Python invocation and on Theta and Titan using the app invocation of the bash script. Depending on the software stack available on the resource, the app function invocation avoids potential conflicts between Swift‘s embedded Python interpreter and the Python used by the deep learning frameworks by setting the PATH, PYTHONPATH, and other environment variables appropriately for the system in question.

## Results

In this section, we measure the performance of the CANDLE/Supervisor system for the cancer pilot workloads. We measure quantities relevant to the performance of a workflow system, namely, system utilization, task start-up latency, and task rate scaling.

### System utilization analysis

In our first test, we measure system utilization on NERSC Cori. This test measures the fraction of the system available to the ML libraries, everything else is treated as overhead. In this test, we used the P:DRUG pilot workflow. The plots that follow illustrate the capability of our hyperparameter optimization infrastructure. Two search approaches, random and model-based searches, were scaled up to 360 nodes.

To perform the CANDLE hyperparameter optimization, we installed the CANDLE/Supervisor environment with EMEWS configured to use mlrMBO as the optimization algorithm. We used the ML package that provides a deep learning environment for Python 2.7, including Keras, TensorFlow, Theano, etc., provided by the NERSC administrators.

**Fig. 2:**
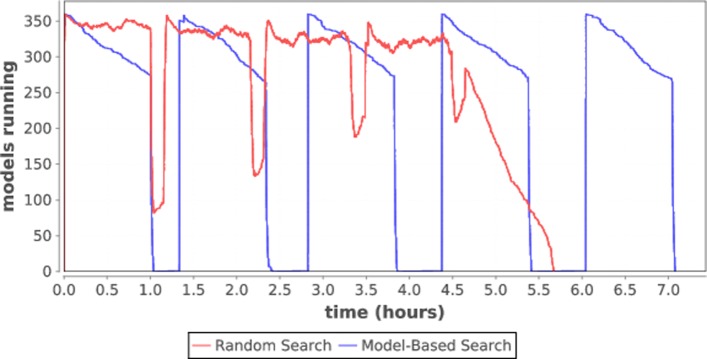
System utilization for hyperparameter optimization on Cori

On Cori, we ran P:RAS and P:TREAT benchmarks on 360 nodes. For P:RAS, we ran two different hyperparameter search strategies: random search and model-based-search, both with a budget of 1800 parameter configurations. In the former, 1800 configurations were generated at random and evaluated by the workflow infrastructure. In the latter, 360 configurations are generated at random and the model-based-search generates 360 configurations at each iteration and evaluated. The results are shown in Fig. 2. Our framework scales well to the total number of nodes in the system; there is negligible ramp-up time.

While the performance results show that random search has better resource utilization over model based search, this is due to the fact that model searches cannot proceed to the next sampling iteration until it finishes evaluating all configurations from the previous iteration. In a more realistic run, the models would run longer (10 or more hours), reducing the impact of the gaps between iterations. Additionally, we plan to overlap runs between iterations as described in “Discussion” section.

### Scaling one iteration

**Fig. 3:**
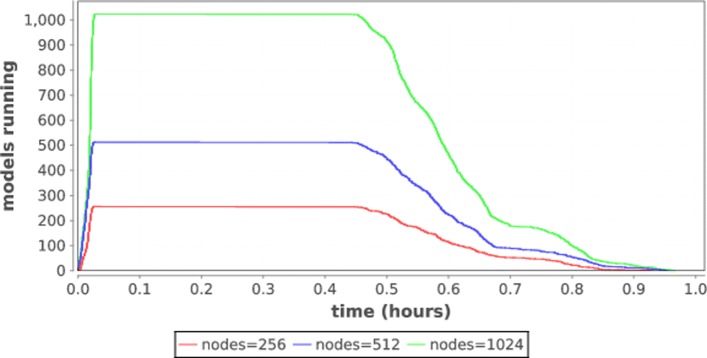
Load profile for increasing workflow scale

In this experiment we run the P:DRUG benchmark with mlrMBO for one iteration at various scales on Titan to determine scalability. For each node count *N*, we recorded the start time and stop time, and plot the number of models running on the system at each point in time. The result is shown in Fig. 3.

As shown in the plot, increasing the number of nodes in the run increases the work done. While there is a considerable impact from task time variability, all tasks exit before they are forced to timeout, which would happen at the 90 minute mark. This shows that the CANDLE/Supervisor system is capable of delivering large-scale computational resources to hyperparameter search workflows.

### Task start-up latency

**Fig. 4:**
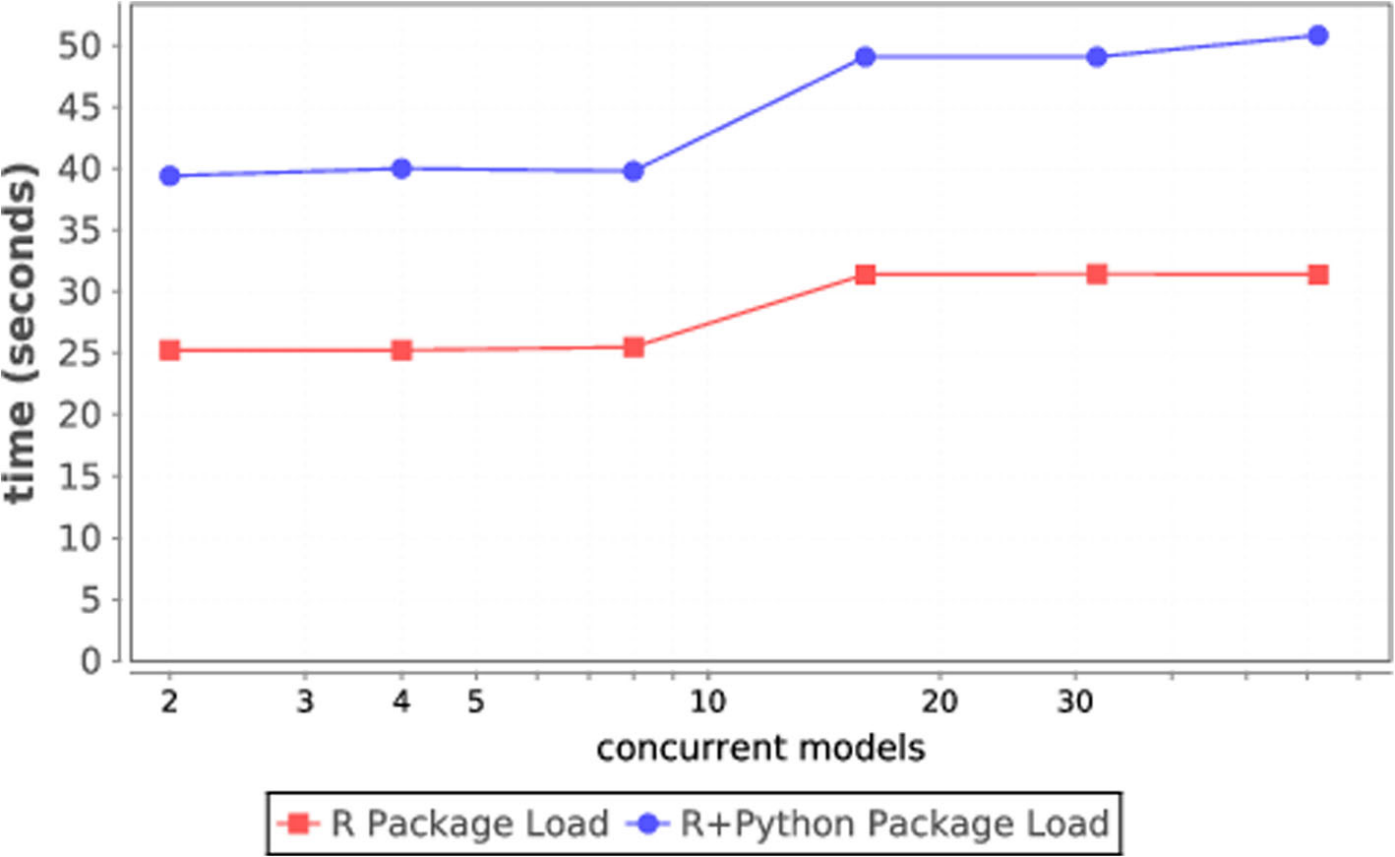
Software load time for Python and R modules on Cori

Our underlying Supervisor workflow engine is capable of quickly distributing tasks to workers, but the workers must load the necessary optimization and ML libraries before executing. The plot in Fig. 4 illustrates this. For increasing workloads (up to 62 nodes, one model per node) on Cori, we profiled the load time for the R packages and Python packages. The total load time is about 50 sec at 62 nodes. We use the in-memory Python and R interpreters available in Swift/T to load these modules, meaning that they are only loaded once per node per workflow, and not for each task.

As shown in the plot, loading the software (not even the training data!) takes almost a minute, even at the modest scale shown. Thus, the ability to keep the modules loaded in the Python and R interpreters from task to task, a unique Swift/T ability, is critical for these workflows.

### Task rate scaling

**Fig. 5:**
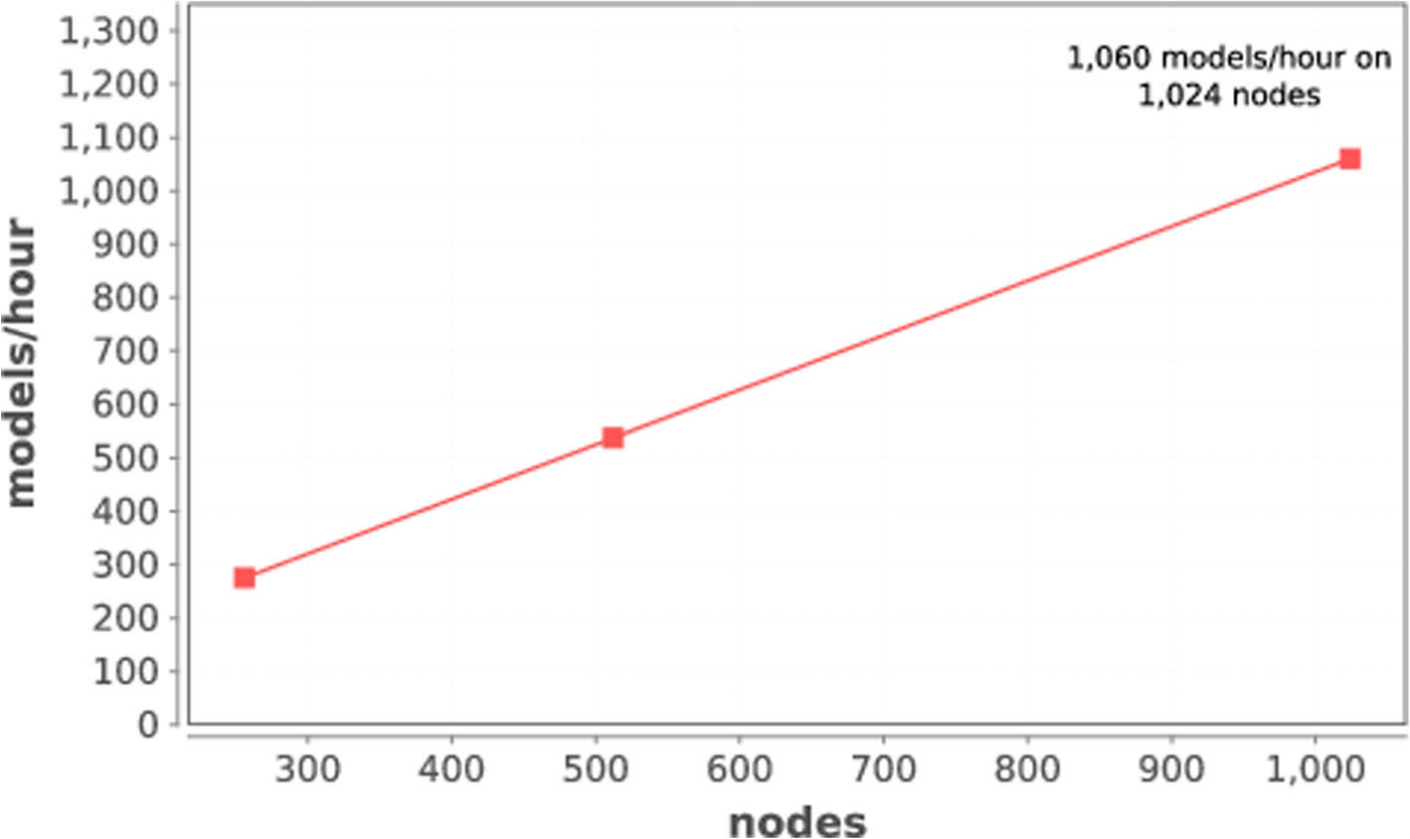
Scalability: models completed per hour on Titan

In this measurement, we seek to summarize the scaling properties of our system by measuring models completed per unit time. In this case, we ran the P:DRUG workflow on Titan at various scale and simply measuring the number of models completed per hour. This result is shown in Fig. 5.

As shown in the plot, the models per hour rate increases linearly up to 1024 nodes, reaching a maximum measured rate of 1060 models/hour. This delivers over 4 petaflops to the deep learning engines used in the workflow (1024 NVIDIA K20X @ 3.95 TF = 4.045 PF, single precision).

## Discussion

This paper demonstrates the basic features of a scalable workflow framework for machine learning applied to problems in cancer research, but there are many additional features yet to investigate and develop.

First, we plan to address the cyclical nature of our workflows and resolve the gap problem shown in Fig. 2. We will modify the optimizers to be “streaming optimizers”, which will be capable of producing more sample points as soon as sample results are available, instead of one iteration at a time. This may take significant modification to existing optimizer codes, but the potential gain in utilization will be worth the effort.

Second, we plan to support larger data-parallel machine learning models in our workflows. Swift/T already has support for parallel MPI jobs, etc. [12] Our workflows will be able to use this feature to dynamically select the resource levels to apply to each model execution.

Third, we are applying our experience using these optimizers to develop new optimizers for hyperparameter optimization. These optimizers will be compatible with the CANDLE/Supervisor framework and we will easily be able to measure their quality against existing techniques on large-scale problems.

## Conclusions

Applying machine learning to cancer research is a promising approach in many aspects, including the benchmark problems used here, the RAS pathway, drug response, and treatment strategies. A significant challenge in this area is selecting and parameterizing the neural network models and software packages to be applied to these problems. In this paper, we described the relevant workflows in some detail. We then offered our solution by presenting CANDLE/Supervisor, a framework for rapidly testing hyperparameter optimization techniques for machine learning models, and showed how it is applied to several cancer benchmarks.

The CANDLE/Supervisor framework offers multiple features to support machine learning in cancer research. First, is has a pluggable architecture, allowing users to easily substitute the optimizer or ML problem. Second, it is efficient, allowing use of large-scale resources, as described in “Results” section. Third, it is portable, and allows researchers to benefit from the abundant computational concurrency available on many leadership-class systems. The software has also been tested on clusters and individual workstations. It is available at https://github.com/ECP-CANDLE.

As the project progresses, the design of the Pilots will evolve, either by modification of the default model paremeters (within a certain class of ML networks) or via construction of new networks, which may in turn necessitate modifications at the Supervisor level. We intend to periodically release updated Pilots, synchronized with appropriate updates at all levels of the CANDLE/Supervisor.

Cancer research is an important topic with significant societal impact. CANDLE/Supervisor allows research teams to leverage the most powerful high-performance computer systems in this problem space.
